# Top-down effect of body representation on pain perception

**DOI:** 10.1371/journal.pone.0268618

**Published:** 2022-05-26

**Authors:** Miki Matsumuro, Ning Ma, Yuki Miura, Fumihisa Shibata, Asako Kimura

**Affiliations:** 1 College of Information Science and Engineering, Ritsumeikan University, Kusatsu, Shiga, Japan; 2 Graduate School of Information Science and Engineering, Ritsumeikan University, Kusatsu, Shiga, Japan; Baylor College of Medicine, UNITED STATES

## Abstract

Many studies on body representation intend to change the perceived size, material, and structure of the body. However, whether the perception of a stimulus can be modified by manipulating body representation remains largely unexplored. Thus, the current study investigated the relationship between transparency of body representation and pain perception. Using augmented reality technology, we made the participants’ limbs transparent and analyzed changes in body representation. Using a questionnaire, we confirmed that the participants perceived their limb as transparent. Simultaneously, their sense of ownership of the limb decreased, because they felt that it no longer belonged to their body. The participants were given an electrical stimulus to assess their subjective perception of pain intensity. An increase in limb opacity decreased the perception of pain, which, in turn, increased the feeling of transparency. These results suggested that the feeling of transparency in their limb favored the decrease in perceived pain. This effect was modified by body ownership, where high levels reinforced the analgesic effect. However, body ownership displayed a positive relationship with perceived pain. The study suggests that body transparency may constitute a strategy for decreasing refractory pain given that body ownership is retained at a high level.

## Introduction

Humans use a mental representation of their body to interact with the world [1–3]. For example, body representation influences reactions to obstacles and decisions to stride over, pass under, or break through them. Although several features of the human body, such as posture and position, are temporal and variable, other features are stable and invariant, such as structure (typically a head, two arms, two legs, and a torso) and body composition. These features play an important role in human perception.

Senna et al. [4] intended to manipulate the perceived material of the body using auditory feedback. The participants listened to a feedback sound of a hammer hitting marble simultaneously with a gentle tapping on their hand. Afterward, the participants reported that their hand was stiffer, heavier, harder, and less sensitive. Additionally, their galvanic skin responses (GSRs) to a needle approaching the hand differed according to the reported feeling. This study demonstrated that a sound feedback can modify the representation of the body material, which subsequently influences physiological responses to a pain threat. The GSR increased when the participants experienced the illusion. However, the needle did not touch the fingers of the participants, which prevented from experiencing pain. Therefore, whether changes in body representation, in fact, influenced pain perception is unclear.

Other studies investigated changes in body representation, especially in terms of size [5–9]. Although many of them included a perceptual task, their main objective was to investigate changes in body representation. For example, several studies estimated the distance between two points on a limb to analyze whether manipulation elongated limb representation. However, none of these studies examined changes in perception that may indirectly be related to a change in body representation, such as the link between hardness and pain perception or between size and weight perception. The study on whether the perception of a stimulus can be modified by manipulating body representation has only started in recent years but especially focused on pain [4, 10–13].

Martini et al., [10] investigated the relationship between skin color and threshold for heat pain. The participants observed a virtual forearm with a different wrist color to replace their biological one. When the virtual forearm was red, the participants felt pain for temperatures less than the one that triggered pain when the arm was blue. However, the authors did not confirm whether the participants felt their limb turn red or blue, that is, body representation. Color may have been associated with the stimulation or with a device attached to the wrist. In addition, using the virtual forearm may interfere with the sense of body ownership, which has been illustrated to influence pain perception, as described in the next section. Notably, the sense of ownership for the virtual arm did not differ among the conditions in the study of Martini et al. [10], which suggests that body representation influenced pain perception independent of the feeling of ownership.

### Body ownership

The sense of body ownership denotes the sensation that an object belongs to one’s body. Humans exhibit a strong feeling of ownership for the biological body, which is extended to non-body parts in certain situations [14–17]. The rubber hand illusion, where participants feel as if a rubber hand belongs to their body, is a famous example of such an extension [18]. Although several results are conflicting, high levels of body ownership of a rubber hand or a virtual limb generally decrease subjective pain intensity [19–21].

The majority of these studies, including the study on skin color conducted by Martini et al. [10], used artificial limbs, such as a rubber hand or a virtual limb. In these situations, information from multimodalities is conflicting. Indeed, the positions of the rubber hand and the biological hand never fully match, which resulted in a conflict between the proprioceptive information from the eyes and the biological hand. Even if the positions of the biological and the virtual limbs are sufficiently adjusted, the virtual limb fails to reproduce the visual features of the biological one or movements signaled by the brain. As a result, the participants should resolve these conflicts during the task, which may influence pain perception [17]. Thus, the current study aimed to investigate the effect of body representation independently from the effect of ownership using augmented reality (AR) technology, where a real-time image of the participants’ limb is manipulated.

### Overview of this study

We modified the representation of body features that are stable and consistent over time and investigated the effect of changes on pain perception. The participants’ forearm and hand (collectively referred to as limb) were made transparent. However, the analgesic effect of transparency remains unclear. Martini et al. [11] illustrated the interactive effect between body ownership and transparency on heat threshold. Only when the virtual limb was the most transparent can a negative correlation be observed between body ownership and heat threshold. However, transparency itself exerts no analgesic effect. Alternatively, Matamala-Gomez et al. [22] conducted a similar study and proposed that increasing transparency decreased pain in patients with complex regional pain syndrome type I.

Thus, the current study hypothesized that limb transparency would decrease perceived pain. The transparent limb may be considered unaffected by an external stimulus, which is a notion similar to invisible humans in fictional stories. This impression may lead to a decreased pain sensation; in this regard, previous studies demonstrated that expectations of pain influenced the physiological response to pain [23]. Additionally, this blunted pain perception may be enhanced by the impression that invisible body parts cannot be touched and, thus, cannot be affected by an external stimulus. To test this hypothesis, we showed participants that an object is passing through their limbs. This manipulation is expected to result in a further decrease in perceived pain.

Martini et al. [11] reported a decreased sense of ownership for transparent limbs. Many studies demonstrated the analgesic effect of observing one’s body or artificial limbs as if they belonged to their body [20, 21]. Therefore, a decrease in body ownership would interfere in the investigation of the effect of body representation. The AR technique in our experiment enabled the observed arm to retain all features of the participants’ limb, even small movements, apart from the alpha value. For this reason, we expect that the participants will retain high levels of body ownership for the observed limb. As such, we investigate the effect of body representation independently from the effect of ownership despite changes in the alpha value. Additionally, we performed a manipulation to recover the decreased feeling of body ownership.

We investigated changes in body representation and body ownership after three cognitive manipulations (detailed in the Manipulations of the Apparatus and materials section) using a questionnaire that assesses the participants’ feeling of their limbs. To acquire a subjective measure of pain intensity, the participants were then given an electrical stimulus. Two experiments were conducted. The second experiment was conducted with a modified manipulation of body ownership, because the sense of transparency and body ownership varied too synchronously during the first experiment, which prevented us from reaching a fair conclusion. Both experimental methods, which were nearly identical, and their results are described.

## Materials and methods

### Participants

The study recruited 21 undergraduate and graduate students (2 females and 19 males) for Experiment 1 and 27 undergraduate and graduate students (2 females and 25 males) for Experiment 2. We did not record the age of each participant but recruited them from students with ages ranging from 20 to 26 years. All participants were right-handed and presented with normal or corrected-to-normal vision. We confirmed that all participants felt tolerable pain after electrical stimulation. The participants remained unaware of the objective of this study and did not undertake related experiments prior to the study.

### Apparatus and material

#### AR system

**Fig 1:**
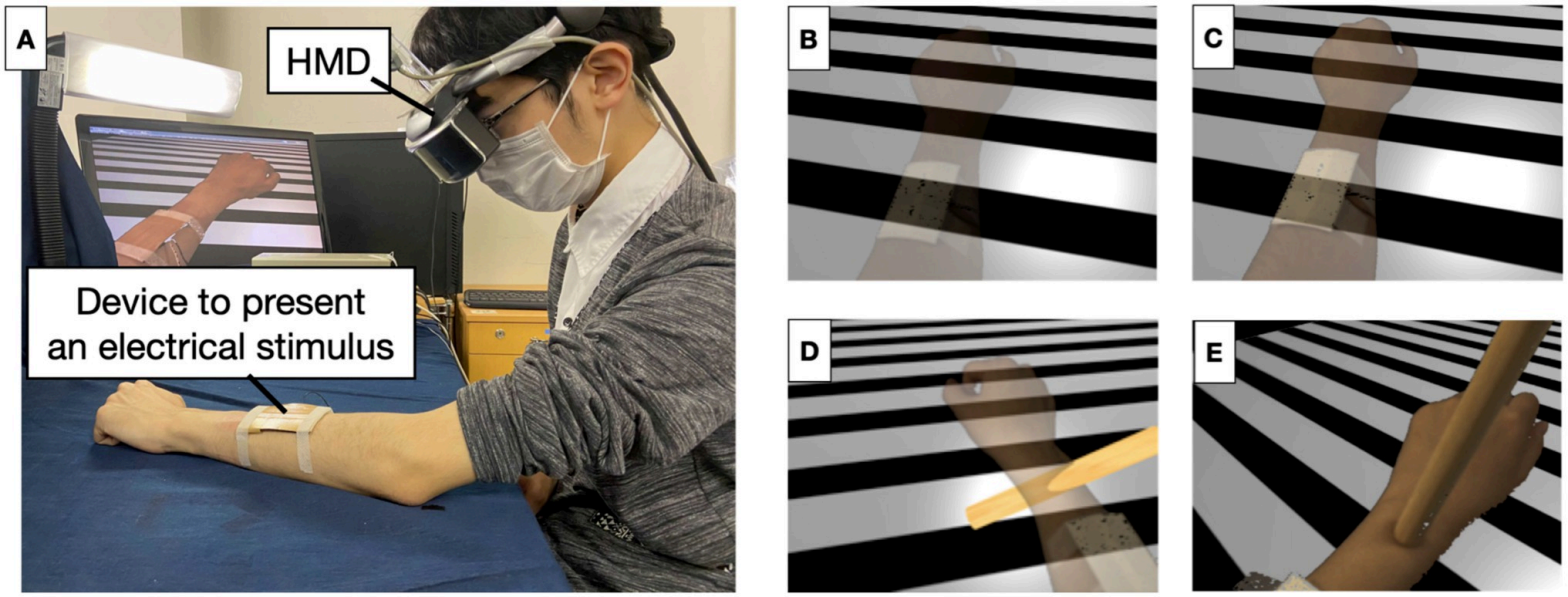
Experimental environment and manipulation of the perception. (A) Experimental set-up. (B) Appearance of the limb on the HMD with the alpha-value set at 0.25. (C) Appearance of the limb on the HMD with the alpha-value set at 0.5. (D) Virtual stick passing through a participant’s limb. The alpha value was 0.25. (E) Touching a participant’s wrist with a real wooden stick with an alpha value of 1.0.

We used an HM-A1 (Canon) video see-through-type head-mounted display (HMD) with a resolution of 1280 × 1024 pixels per eye and a total field of view of 41° horizontally and 31° vertically displayed at 60 Hz. We implemented the AR environment using the AR platform system (Canon, MP-110). Head tracking was performed with Polhemus 3 SPACE FASTRAK. Fig 1A presents the experimental set-up.

#### Manipulations

We manipulated the opacity of the participants’ limbs to alter their body representation. Toward this end, the scene was acquired from the participants’ perspective with the camera on the HMD, whereas the limb was extracted from the scene based on its color. We set the value of the alpha channel of the extracted area to 0.25 in Experiment 1. However, the level of body ownership decreased greater than we expected according to the change in the alpha value. Therefore, we used 0.50 for the alpha channel in Experiment 2. The limb was presented on a virtual black-and-white background (Fig 1B and 1C). In the condition where the alpha value was changed, the alpha value was decreased from 1 to 0.50 or 0.25 over 2 to 3 s after a cue from the investigator. The participants observed the change, which permitted them to conceive that their limb was transforming.

Two additional manipulations were used to alter the sense of body ownership and enforce the intangible nature of body representation. First, we used the pass-through manipulation, where the participants observed a virtual stick passing through their limb (Fig 1D). This step increased the feeling that the limb is intangible. First, we showed the participants the virtual stick and confirmed that they observed it. We moved a virtual stick through the participant’s wrist from upper to lower and from lower to upper and repeated this process five times. The investigator carefully adjusted the position of his/her hand to avoid displaying it on the HMD.

The second manipulation aimed to recover the decreased level of body ownership. In Experiment 1, the participants were instructed to move a finger and observe it (movement manipulation). Previous studies demonstrated that visuo-motor consistency can increase the feeling of body ownership of the observed limb [24]. First, the participants bent their wrist backward to avoid touching the desk when moving their fingers. The experimenter then called out the name of one of the fingers, such as the index finger, in random order. In response, the participants bent the named finger toward their palm at the metacarpophalangeal joint and placed it back to the normal position. Each finger was called twice in random order for each participant. Finally, they returned their wrist angle to straight.

The results for Experiment 1 indicated that the movement manipulation was unable to recover the body ownership of the participants. In Experiment 2, we used the touch manipulation, where the participants’ wrist was touched 10 times using a real wooden stick (Fig 1E). The participants were instructed to continue observing the limb and as it was being touched by the stick. Visuo-tactile synchronous stimulation is a very popular method for increasing the sense of body ownership [18, 25]. We adjusted the range of the color extraction to enable the observation of the stick. The experimenter held the stick carefully so that his hand was not displayed on the HMD.

We omitted the abovementioned manipulations from the following conditions: alpha = 1.0 and the without the addition of each manipulation. No other alternative task or observation time was prepared. The time duration for observing the limb and the observing material (the wooden and virtual sticks) differed across conditions. The participants were allotted 10 s to observe the (manipulated) limb before filling out the questionnaire or receiving the pain stimulus under all conditions.

We investigated eight conditions, namely, 2 (alpha value = 1 or 0.25) × 2 (with or without finger movement) × 2 (with or without pass-through) in Experiment 1. In Experiment 2, wrist touch replaced the finger movement as follows: 2 (alpha value = 1 or 0.5) × 2 (with or without wrist touch) × 2 (with or without pass-through).

#### Electrical stimulation

We used an electrical stimulus to induce pain, because we targeted the analgesic effect in case of brief acute painful sensations, such as the sting of a needle (e.g., injection). The stimulus was a direct current boosted by a Cockcroft–Walton circuit and controlled by a laptop computer through an input/output board (RBIO-2U; Kyohritsu Electronic Industry Co., Ltd.). We then fixed a conductor (diameter: 0.12 mm, 10 cores) to a 1-mm thick rubber sheet and applied the current to the conductor in order to trigger pain. The intensity of the electrical stimulation was 320 V at a current of 1.8 mA, which was generally deemed tolerable, with a pulse width of 0.15 s. We used a fixed intensity to avoid multiple stimulations for determining a subjectively equivalent point. We attached the device at the midpoint between the ulnar styloid and the epicondylus medialis on the radial side of the forearm.

#### Questionnaire

We collected data on body representation using a questionnaire. Table 1 presents the items, which included those based on previous works and our pilot study. In the latter, the participants who did not participate in Experiment 1 or 2 were asked about how they felt about their limb at various alpha values. Based on the answers, we included items about transparency and intangibility. We treated the two constructs separately, because we expected that transparency and intangibility would decrease perceived pain more than only the transparency feeling is high.

**Table 1:** Items in the questionnaire.

Number	Item
Ownership	
3	*My arm seems to be present in the environment*
6	I feel as if the observed arm were not mine (R)
11	*I feel as if the observed arm is a real one*
16	The observed arm looks like mine
19	The observed arm does not look like mine (R)
Transparency	
2	My arm feels sparse
9	I feel as if my arm were transparent
20	The arm is transparent
Intangibility	
5	I feel as if something could pass through my arm
12	*I feel as if I were a ghost*
18	I feel as if my arm were empty
Anxiety	
8	I feel anxious when observing the arm
10	I feel ill when observing the arm
13	I feel calm when observing the arm (R)
15	I feel fear when observing the arm
Stiffness	
1	My arm feels stronger
4	My arm feels harder
7	My arm feels sensitive (R)
14	My arm feels numb
17	My arm feels heavier

*Note*. We excluded items written in italic. R = inverted scale. Items were shown for each feeling. The item number indicates the order of each item in the questionnaire.

Items 9 and 20 directly asked about the feeling of their limb becoming transparent. Item 2 pertained to the sparsity of the limb. If an object’s density is sparse, we can see the scene behind it identical to a transparent object. Items on intangibility focused on the feeling that the limb could not be touched: pass through (item 5) and empty (item 18). As representative instance of an object that cannot be touched, item 12 “*I feel as if I were a ghost*” was added.

As previously described, previous studies demonstrated that the sense of body ownership influenced the subjective perception of pain intensity. Thus, we assessed their level of ownership over an observed arm using items from Botvinick and Cohen [18] and Kalckert and Ehrsson [26] (items 6, 16, and 19). We replaced the term “rubber hand” with “observed arm.” We also added items to assess the perception of the reality of the observed limb (items 3 and 11).

Anxiety is also known as a feeling that increases the intensity of perceived pain and decreases pain threshold [27, 28]. To measure anxiety, we used several terms from the Anxiety Sensitivity Index [29] and State–Trait Anxiety Inventory [30]: anxious, ill (antonym of comfortable), calm (inverted scale), and fear (antonym of calm and secure). Items 8, 10, 13, and 15 evaluated how strongly each construct was perceived upon observing the manipulated limb.

Additionally, Senna et al. [4] demonstrated that feelings of hardness and heaviness of the body, which are described as stiffness similar to stone, influenced pain perception. We adapted items from the said study on hardness, sensitivity (inverted scale), numbness, and heaviness (items 4, 7, 14, and 17, respectively). Item 1 pertains to the strength of the limb and was added, because a stone (marble) is stronger than human arms. We named the construct that represented these items as “stiffness” after Senna et al. [4].

All items were prepared in the Japanese language. Items were rated using a seven-point Likert-type scale (from 1: not at all to 7: extremely). The order of items was indicated in Table 1.

### Procedure

Both Experiments 1 and 2 were conducted in two phases, namely, questionnaire survey and pain evaluation. Before the start of the two phases, the participants were briefed about the objective and details of the experiment and provided informed written consent. After that, the investigator marked the location on which to attach the device for electrical stimulation. The device was attached at the start of each phase and detached during rest. Afterward, the participants wore the HMD, which initiated the questionnaire phase. The participants answered the questionnaire under eight conditions. After sufficient rest, the pain evaluation phase started, and perceived pain was assessed in all eight conditions. We describe the procedure in each phase below.

#### Questionnaire phase

In this phase, a questionnaire was used to investigate body representation. The participants wore the HMD and placed the non-dominant forearm on a desk (Fig 1A). At this point, all participants observed their forearm with an alpha value of 1.0 under all conditions. Afterward, the manipulations were conducted according to the condition of the trial. In Experiment 1, the order of manipulations is as follows: change in alpha value, pass-through manipulation, and movement manipulation. We followed this sequence because pass-through manipulation may influence the sense of body ownership. In Experiment 2, the touch manipulation was followed by the alpha-value manipulation, whereas the pass-through manipulation was performed at the last. We used this sequence to avoid conflict between the touch and pass-through manipulations.

After all manipulations, the participants observed their (manipulated) forearm for 10 s and answered the questionnaire. Table 1 presents the questionnaire items. We repeated this procedure for all conditions, which resulted in eight evaluations for each participant. The order of the conditions was randomized.

#### Pain evaluation phase

Pain evaluation was conducted after a more than five-minute rest. At the start of the pain evaluation phase, we verified whether the participants felt pain with the electrical stimulation. The participants observed their limbs without the HMD and were given the electric stimulus, which is the same as that used in the experiment. If the participants reported that they did not feel pain, then the experiment was terminated and he/she was excluded from the study. In Experiment 2, the participants also evaluated pain intensity induced by this test stimulation using the same method for pain evaluation, which was used as the baseline evaluation (for details, see the Analysis section).

In the evaluation, the procedure was identical to that in the questionnaire phase up to the moment where the participants observed their manipulated limb. Before the HMD shut down, an electrical stimulus was applied simultaneously with a verbal cue. The participants removed the HMD and assessed pain intensity using the visual analog scale. We prepared a 100-mm line with the left and right parts representing “no pain” and the “worst possible pain.” An x was used to mark the point that reflects the level of perceived pain. All the participants experienced all conditions once in random order with a rest of approximately 2 min between trials.

Both phases were completed in one session of 90 min.

#### Ethical approval

The Ethics Review Committee for Medical and Health Research Involving Human Subjects of the Ritsumeikan University approved the study (BKC-人医-2018-033). The individual in this manuscript has given written informed consent (as outlined in PLOS consent form) to publish these case details and their images (Fig 1) alongside this manuscript.

### Analysis

Prior to data analysis, we confirmed that invalid items were excluded from the questionnaire and calculated the scores for five feelings (i.e., ownership, transparency, intangibility, anxiety, and stiffness) and pain. The scores for all feelings were the average score of items score (1: not at all to 7: extremely) prepared for each feeling. To calculate the pain score, we first measured the distance from the left-most of the VAS to the x-mark for each condition. Then the difference between the distance in each condition and the distance in the baseline evaluations was divided by the distance in the baseline evaluation. In Experiment 1, baseline evaluation was defined as the condition without manipulation (i.e., the alpha value was 1.0 without the movement and pass-through manipulations). It was one of the conditions of Experiment 1 and measured using the HMD. In Experiment 2, baseline pain evaluation preceded the pain evaluation in all conditions and was measured without the HMD (see the Procedure section for details), to reflect the participants’ sensitivity for pain more accurately. In both experiments, when perceived pain was equal to the baseline, the score became 0.

To investigate the effect of each manipulation on each feeling and pain perception, we conducted 2 × 2 × 2 within-subject ANOVA for each score. Experiment 1 included the following factors: alpha-value (1.0 and 0.25), pass-through (with and without pass-through), and movement (with and without movement) manipulations. In Experiment 2, touch manipulation replaced the movement manipulation. The three factors for Experiment 2 were the alpha-value (1.0 and 0.5), pass-through (with and without pass-through), and touch (with and without touch) manipulations. By comparing the effect of the manipulations on the scores for each feeling and the pain score, we investigated which feeling covaried with the intensity of perceived pain. In addition, we analyzed a repeated measures correlation between each feeling and pain score to investigate which feeling is most related to perceived pain.

The results of ANOVAs are presented as follows. The significant level was set to 0.05. If a three-way interaction was significant, then we conducted follow-up analyses and presented the results in a table. In this case, if a two-way interaction is significant, then we omitted the descriptions of its follow-up analyses. Additionally, if a two- and/or three-way interaction was significant, then we omitted the description of the direction of the main effect of the factors included in the interaction. We performed post hoc analysis using Shaffer’s method and designated a generalized eta squared ($\eta_{G}^{2}$) to represent the effect size of ANOVAs.

## Results

### Validity of questionnaire items

Prior to analysis of the questionnaire scores, we reconsidered the validity of the relationship between each item and its related feeling. After deliberation among the researchers, the following items were excluded from the questionnaire (italicized items in Table 1). The first is item 3 “My arm seems to be present in the environment,” which pertains to the perception of the presence of the arm. In Japanese, an adjective that expresses someone who has less presence is identical to sparse (“薄い usui”) in item 2. Along with the order of these items, the response to item 3 may have been influenced by the response to item 2. For this reason, we excluded this item from the analysis. We retained item 2 as the item for transparency because when people see the word sparse (“薄い”) without any special context, they do not perceive it as related to the feeling of existence.

In addition, we removed item 12 “I feel as if I were a ghost.” Typically, ghosts are considered transparent and intangible. Therefore, although we intended to use this item for measuring intangibility, both transparent and intangible feelings would be included in the response to this item. Lastly, item 11, which evaluated the perception of the reality of the limb, was excluded. Although a real object can increase body ownership over it, this condition is neither necessary nor sufficient. Synchronous visuomotor information could induce the feeling of ownership even over a robot hand [24].

The subsequent text discusses the results of changes in mental representation and pain for Experiments 1 and 2.

### Results of Experiment 1

#### Modification of body representation

**Fig 2:**
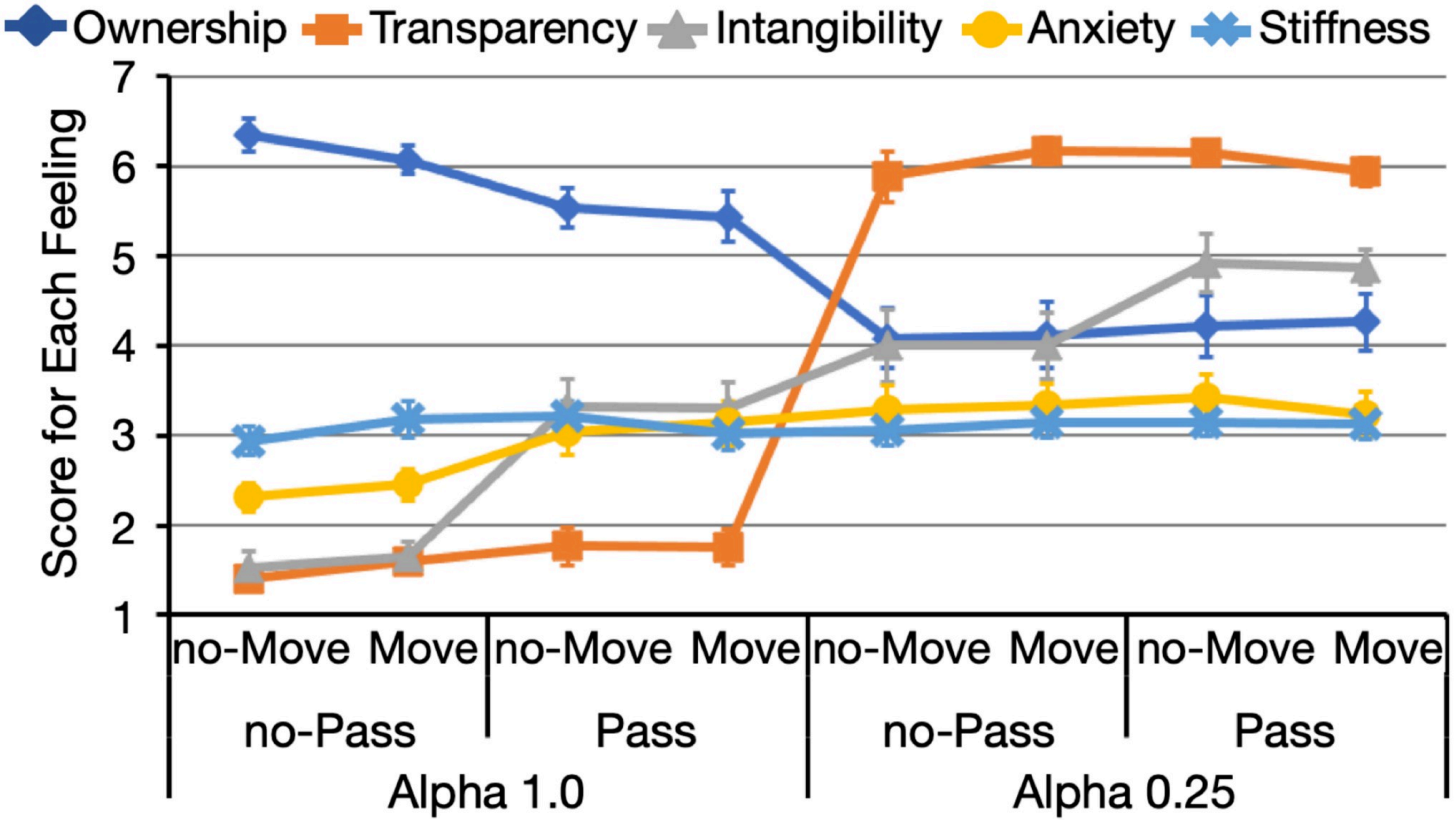
Averaged scores for each feeling and each condition in Experiment 1 (*n* = 20). Each point is the average score of all items for each feeling. On the x-axis, data are grouped according to the alpha-value (1.0 or 0.25), pass-through (with or without pass-through), and movement (with or without movement) manipulations. Error bars represent the standard errors.

Data from one participant who did not complete all conditions were excluded from the following analyses. Fig 2 displays the changes in the scores for each feeling and each condition in Experiment 1. We conducted a 2 (alpha value) × 2 (movement manipulation) × 2 (pass-through manipulation) ANOVA. The results indicate that the three-way interaction was non-significant for any feeling (*F*s (1, 19) < 1.425, *p*s > 0.248, $\eta_{G}^{2}s<0.003$).

*Ownership*. The two-way interaction between the alpha-value and pass-through manipulations was significant (*F*(1, 19) = 14.696, *p* = 0.001, $\eta_{G}^{2}=0.029$). The decrease in alpha value decreased the ownership score for the pass (*F*(1, 19) = 13.460, *p* = 0.002, $\eta_{G}^{2}=0.188$) and no-pass (*F*(1, 19) = 39.268, *p* < 0.001, $\eta_{G}^{2}=0.433$) conditions. Moreover, the pass-through manipulation decreased the ownership score only at an alpha value of 1.0 (alpha value = 1.0: *F*(1, 19) = 11.739, *p* = 0.003, $\eta_{G}^{2}=0.126$; alpha value = 0.25: *F*(1, 19) = 0.609, *p* = 0.445, $\eta_{G}^{2}=0.002$). The main effect of the alpha value was significant (*F*(1, 19) = 27.753, *p* < 0.001, $\eta_{G}^{2}=0.310$). No other interaction or main effect was significant (*F*s (1, 19) < 3.265, *p*s > 0.085, $\eta_{G}^{2}s<0.015$).

*Transparency*. Only the main effect of the change in alpha value was significant (*F*(1, 19) = 355.627, *p* < 0.001, $\eta_{G}^{2}=0.888$). As the alpha value decreased, the participants experienced a stronger feeling of transparency. The other effects and interactions were non-significant (*F*(1, 19) < 3.450, *p*s > 0.075, $\eta_{G}^{2}s<0.006$).

*Intangibility*. The interaction between the alpha-value and pass-through manipulations was significant (*F*(1, 19) = 5.794, *p* = 0.026, $\eta_{G}^{2}=0.026$). The small alpha value significantly increased the intangibility score regardless of the pass-through manipulation (*F*(1, 19) > 29.000, *p*s < 0.001, $\eta_{G}^{2}s>0.295$). Moreover, the pass-through manipulation increased intangibility less at an alpha value of was 0.25 (*F*(1, 19) = 12.865, *p* = 0.002, $\eta_{G}^{2}=0.088$) compared with 1.0 (*F*(1, 19) = 46.894, *p* < 0.001, $\eta_{G}^{2}=0.403$). The score for intangibility resulted in a significant main effect on the alpha-value (*F*(1, 19) = 48.063, *p* < 0.001, $\eta_{G}^{2}=0.385$) and pass-through (*F*(1, 19) = 50.905, *p* < 0.001, $\eta_{G}^{2}=0.212$) manipulation. No other main effect or interaction reached significance (*F*(1, 19) < 0.125, *p*s > 0.730, $\eta_{G}^{2}s<0.001$).

*Anxiety*. A similar tendency was observed for anxiety in terms of the interaction between the alpha-value and pass-through (*F*(1, 19) = 13.050, *p* = 0.002, $\eta_{G}^{2}=0.029$) manipulations, which is significant, as well as the main effect of the two factors (alpha-value manipulation: *F*(1, 19) = 17.406, *p* < 0.001, $\eta_{G}^{2}=0.077$; pass-through manipulation: *F*(1, 19) = 7.140, *p* = 0.015, $\eta_{G}^{2}=0.031$). However, the simple main effect of the alpha-value manipulation differed from that of intangibility, that is, alpha value = 0.25 increased anxiety only without pass-through manipulation (*F*(1, 19) = 23.102, *p* < 0.001, $\eta_{G}^{2}=0.204$). With the pass-through manipulation, the score remained high and uninfluenced by the alpha value (*F*(1, 19) = 2.801, *p* = 0.111, $\eta_{G}^{2}=0.012$). The pass-through manipulation increased anxiety only at alpha value = 1.0 (*F*(1, 19) = 14.363, *p* = 0.001, $\eta_{G}^{2}=0.128$) and not at alpha value = 0.25 (*F*(1, 19) = 0.008, *p* = 0.930, $\eta_{G}^{2}<0.001$). We found no significant main effect or interaction (*F*s (1, 19) < 2.190, *p*s > 0.155, $\eta_{G}^{2}s<0.003$).

*Stiffness*. No interaction or main effect was significant for the stiffness score (*F*s (1, 19) < 2.560, *p*s > 0.125, $\eta_{G}^{2}s<0.009$).

These results indicate that the change in alpha values largely influenced nearly all feelings, with the strongest effects observed for transparency ($\eta_{G}^{2}=0.888$; others: $\eta_{G}^{2}>0.385$). The alpha-value and pass-through manipulations influenced the scores for ownership, intangibility, and anxiety. The decrease in alpha value also decreased the ownership score, whereas the pass-through manipulation decreased the ownership score at alpha value = 1.0. The intangibility score increased with the decrease in alpha value, where the pass-through manipulation further increased intangibility. The anxiety score at alpha value = 1.0 and without pass-through manipulation was less than those under the other conditions.

#### Pain

**Fig 3:**
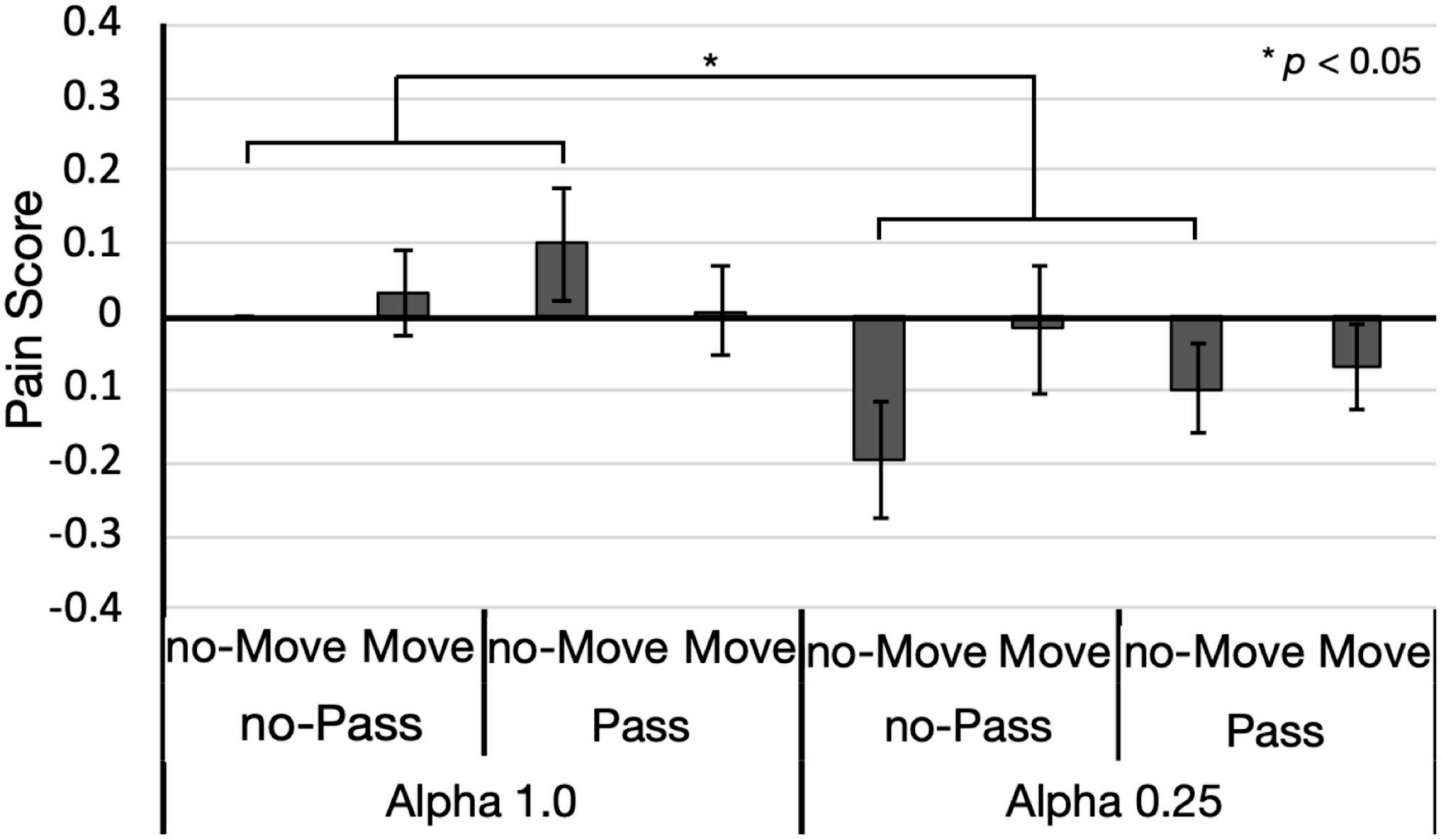
Pain scores under each condition of Experiment 1 (*n* = 20). Each bar represents the average pain score. When the evaluation was identical to baseline, the pain score reached 0. Baseline evaluation was conducted without manipulation (i.e., the left-most bar in the graph). A negative score indicates that perceived pain decreased than the baseline evaluation, whereas a positive score denotes a score for perceived pain that exceeded that at baseline evaluation. On the x-axis, data are grouped according to alpha-value (1.0 or 0.25), pass-through (with and without pass-through), and movement (with and without movement) manipulations. Error bars constitute standard errors.

Fig 3 depicts the pain scores under each condition. If the intensity of perceived pain was identical to that obtained under the without manipulation condition (i.e., alpha value = 1.0 without the pass-through and movement manipulations), the score reached 0. We performed a 2 (alpha value) × 2 (move manipulation) × 2 (pass-through manipulation) ANOVA on the pain score. The three-way interaction did not reach significance (*F*(1, 19) = 0.064, *p* = 0.803, $\eta_{G}^{2}<0.001$), whereas the two-way interaction between the alpha-value and movement manipulations was significant (*F*(1, 19) = 5.151, *p* = 0.035, $\eta_{G}^{2}=0.014$). Alpha value = 0.25 significantly decreased the pain score when the participants did not move their finger (with movement manipulation: *F*(1, 19) = 1.827, *p* = 0.192, $\eta_{G}^{2}=0.011$; without movement manipulation: *F*(1, 19) = 12.991, *p* = 0.002, $\eta_{G}^{2}=0.116$). The movement manipulation did not exhibit a significant effect regardless of alpha value (*F*s (1, 19) < 3.655, *p*s > 0.070, $\eta_{G}^{2}s<0.028$). The main effect of the alpha value was significant (*F*(1, 19) = 9.984, *p* = 0.005, $\eta_{G}^{2}=0.049$), whereas those of the two other manipulations (*F*s (1, 19) < 0.940, *p*s > 0.345, $\eta_{G}^{2}s<0.005$) and two-way interactions were statistically non-significant (*F*s (1, 19) < 2.651, *p*s > 0.115, $\eta_{G}^{2}s<0.015$).

Analyses of the questionnaire score did not demonstrate a significant interaction between the alpha-value and movement manipulations. However, pain perception seemed to be the most affected when modifying the alpha value (Fig 3). Therefore, transparency may be the feeling that is most related to perceived pain.

We calculated the repeated measures correlation between the scores for each feeling and the pain score. Table 2 displays the correlation coefficients for all combinations. A significantly positive correlation between body ownership and pain score was detected (*r*(139) = 0.198, *p* = 0.019), whereas a significantly negative correlation was noted between transparency and the pain score (*r*(139) = −0.280, *p* < 0.001). The strongest correlation was obtained between transparency and pain score. The analysis also revealed a correlation between the majority of feelings, with a particularly strong correlation between body ownership and transparency (*r*(139) = −0.707, *p* < 0.001).

These results suggested a decrease in perceived pain and an increase in transparency. However, examining the effect of transparency independently from body ownership was difficult, because the alpha-value manipulation exhibited a strong effect on both feelings. Then, we modified the experimental set-up in Experiment 2 to reveal differences in the scores for body ownership with alpha-value manipulation.

**Table 2:** Correlation coefficients between the feelings and pain score in Experiment 1 (*n* = 20).

	Ownership	Transparency	Intangibility	Anxiety	Stiffness
Transparency	−0.707***				
Intangibility	−0.504***	0.664***			
Anxiety	−0.473***	0.401***	0.496***		
Stiffness	−0.040	0.069	0.083	0.072	
Pain	0.198*	−0.280***	−0.130	−0.142	−0.052

* *p* < 0.05.

** *p* < 0.01.

*** *p* < 0.005.

### Results of Experiment 2

#### Modification of body representation

**Fig 4:**
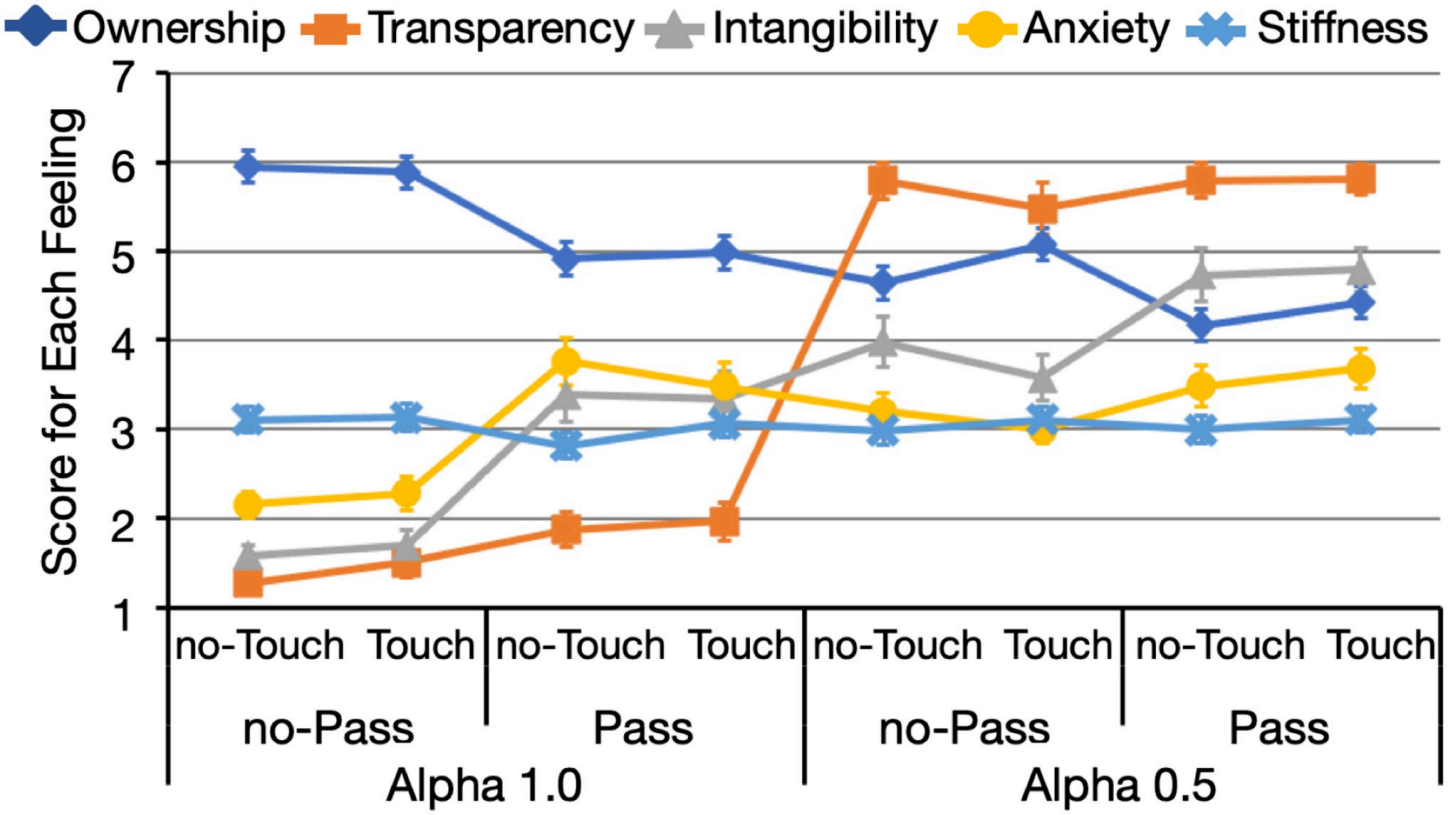
Averaged scores for each feeling and each condition in Experiment 2 (*n* = 26). Each point is the average score of all items for each feeling. On the x-axis, data are grouped according to alpha-value (1.0 or 0.5), pass-through (with or without pass-through), and touch (with or without touch) manipulations. Error bars represent standard errors.

Fig 4 indicates the changes in the scores for each feeling and each condition in Experiment 2. In summary, changes in the alpha-value and pass-through manipulations nearly equally influenced the scores for ownership and intangibility. The alpha value exhibited a very strong effect on transparency, which was also slightly influenced by the pass-through manipulation. Each manipulation influenced anxiety in a complex manner. In general, the participants felt anxious when the alpha value was set to 0.5. At alpha value = 1.0, the pass-through manipulation increased anxiety. No manipulation exerted a significant effect on stiffness.

*Ownership*. The main effects of the alpha-value (*F*(1, 25) = 36.056, *p* < 0.001, $\eta_{G}^{2}=0.124$) and pass-through (*F*(1, 25) = 17.890, *p* < 0.001, $\eta_{G}^{2}=0.102$) manipulations were significant. The decrease in alpha value and the addition of the pass-through manipulation decreased the score for ownership. No other significant main effect or interaction was detected (*F*s (1, 25) < 2.950, *p*s > 0.095, $\eta_{G}^{2}s<0.008$).

*Transparency*. The three-way interaction was non-significant (*F*(1, 25) = 1.391, *p* = 0.249, $\eta_{G}^{2}=0.004$). The interaction between the alpha-value and touch manipulations reached significance (*F*(1, 25) = 4.254, *p* = 0.0497, $\eta_{G}^{2}=0.006$). Although the simple main effect of the alpha value was smaller with the touch manipulation, the decrease of the alpha value greatly increased the score for transparency with (*F*(1, 25) = 221.068, *p* < 0.001, $\eta_{G}^{2}=0.767$) and without (*F*(1, 25) = 361.890, *p* < 0.001, $\eta_{G}^{2}=0.850$) the touch manipulation. The effect of the touch manipulation was non-significant for both alpha-value conditions (*F*s (1, 25) < 1.300, *p*s > 0.270, $\eta_{G}^{2}s<0.009$). Moreover, the main effects of the alpha-value (*F*(1, 25) = 309.560, *p* < 0.001, $\eta_{G}^{2}=0.809$) and pass-through (*F*(1, 25) = 8.774, *p* = 0.007, $\eta_{G}^{2}=0.030$) manipulations were significant. All other effects were non-significant (*F*s (1, 25) < 4.145, *p*s > 0.052, $\eta_{G}^{2}s<0.009$).

*Intangibility*. The three-way interaction did not reach significance (*F*(1, 25) = 2.874, *p* = 0.103, $\eta_{G}^{2}=0.004$), whereas the interaction between the alpha-value and pass-through manipulations was significant (*F*(1, 25) = 7.462, *p* = 0.011, $\eta_{G}^{2}=0.021$). The pass-through manipulation increased the score for intangibility significantly regardless of alpha value, although its effect was smaller at alpha value = 0.5 (*F*(1, 25) = 28.432, *p* < 0.001, $\eta_{G}^{2}=0.115$) than alpha value = 1.0 (*F*(1, 25) = 43.039, *p* < 0.001, $\eta_{G}^{2}=0.347$). The effect of the alpha value was smaller when associated with the pass-through manipulation, but the decrease in alpha value significantly increased the scores for intangibility with (*F*(1, 25) = 17.636, *p* < 0.001, $\eta_{G}^{2}=0.191$) and without (*F*(1, 25) = 60.501, *p* < 0.001, $\eta_{G}^{2}=0.490$) the pass-through manipulation. The significant main effect indicated that the alpha-value (*F*(1, 25) = 42.149, *p* < 0.001, $\eta_{G}^{2}=0.325$) and pass-through (*F*(1, 25) = 55.921, *p* < 0.001, $\eta_{G}^{2}=0.220$) manipulations influenced the score for intangibility. The main effect of the touch manipulation and other interactions were non-significant (*F*s (1, 25) < 2.875, *p*s > 0.102, $\eta_{G}^{2}s<0.004$).

*Anxiety*. The three-way interaction was significant (*F*(1, 25) = 11.075, *p* = 0.003, $\eta_{G}^{2}=0.009$). Table 3 summarizes the results of the follow-up analyses. In general, the score for anxiety remained high with the pass-through manipulation. In contrast, however, this score remained high at alpha value = 0.5 without the pass-through manipulation. At alpha value = 1.0 without the pass-through manipulation, the score for anxiety was lower than those observed under other conditions. The effect of the touch manipulation was small: at alpha value = 0.5, the pass-through manipulation did not increase the anxiety score without the addition of the touch manipulation.

**Table 3:** Results of follow-up analyses for the three-way interaction in anxiety in Experiment 2.

Condition	Analysis		Direction	*F*	*p*	$G_{\eta }^{2}$
alpha 1.0	interaction between touch and pass simple main effect			**7.500**	**0.011**	**0.008**
	effect of touch in	no-pass		0.844	0.367	0.006
		pass		3.600	0.069	0.011
	effect of pass in	no-touch	no-pass < pass	**40.733**	<**0.001**	**0.362**
		touch	no-pass < pass	**31.298**	<**0.001**	**0.216**
	main effect of	touch		0.405	0.530	0.001
		pass	no-pass < pass	**39.981**	<**0.001**	**0.287**
alpha 0.5	interaction between touch and pass			4.218	0.051	0.010
	main effect of	touch		0.005	0.947	<0.001
		pass	no-pass < pass	**19.332**	<**0.001**	**0.053**
no-touch	interaction between alpha and pass simple main effect			**20.915**	<**0.001**	**0.086**
	effect of alpha in	no-pass	1.0 < 0.5	**26.565**	<**0.001**	**0.248**
		pass		1.628	0.214	0.013
	effect of pass in	alpha 1.0	no-pass < pass	**40.733**	<**0.001**	**0.362**
		alpha 0.5		3.417	0.076	0.016
	main effect of	alpha	1.0 < 0.5	**6.293**	**0.019**	**0.031**
		pass	no-pass < pass	**40.394**	<**0.001**	**0.160**
touch	interaction between alpha and pass simple main effect			**6.808**	**0.015**	**0.015**
	effect of alpha in	no-pass	1.0 < 0.5	**13.317**	**0.001**	**0.157**
		pass		0.902	0.352	0.006
	effect of pass in	alpha 1.0	no-pass < pass	**31.298**	<**0.001**	**0.216**
		alpha 0.5	no-pass < pass	**22.744**	<**0.001**	**0.117**
	main effect of	alpha	1.0 < 0.5	**6.895**	**0.015**	**0.045**
		pass	no-pass < pass	**37.907**	<**0.001**	**0.168**
no-pass	interaction between alpha and pass			3.237	0.084	0.009
	main effect of	alpha	1.0 < 0.5	**25.021**	<**0.001**	**0.203**
		touch		0.145	0.706	<0.001
pass	interaction between alpha and touch simple main effect			**4.575**	**0.042**	**0.009**
	effect of alpha in	no-touch		1.628	0.214	0.013
		touch		0.902	0.352	0.006
	effect of pass in	alpha 1.0		3.600	0.069	0.011
		alpha 0.5		1.055	0.314	0.007
	main effect of	alpha		0.058	0.812	<0.001
		touch		0.116	0.737	<0.001

The significant results (*F*, *p*, and $\eta_{G}^{2}$) are presented in **bold font**.

The interaction between the alpha-value and pass-through manipulations (*F*(1, 25) = 18.065, *p* < 0.001, $\eta_{G}^{2}=0.045$) and their main effect (*F*(1, 25) = 8.834, *p* = 0.007, $\eta_{G}^{2}=0.037$ and *F*(1, 25) = 47.148, *p* < 0.001, $\eta_{G}^{2}=0.164$, respectively) reached significance, whereas the opposite is true for the others manipulations (*F*s (1, 25) < 0.176, *p*s > 0.678, $\eta_{G}^{2}s<0.001$).

*Stiffness*. Similar to the result of Experiment 1, no significant interaction or main effect was observed in the score for stiffness (*F*s (1, 25) < 3.915, *p*s > 0.059, $\eta_{G}^{2}s<0.010$).

#### Pain

**Fig 5:**
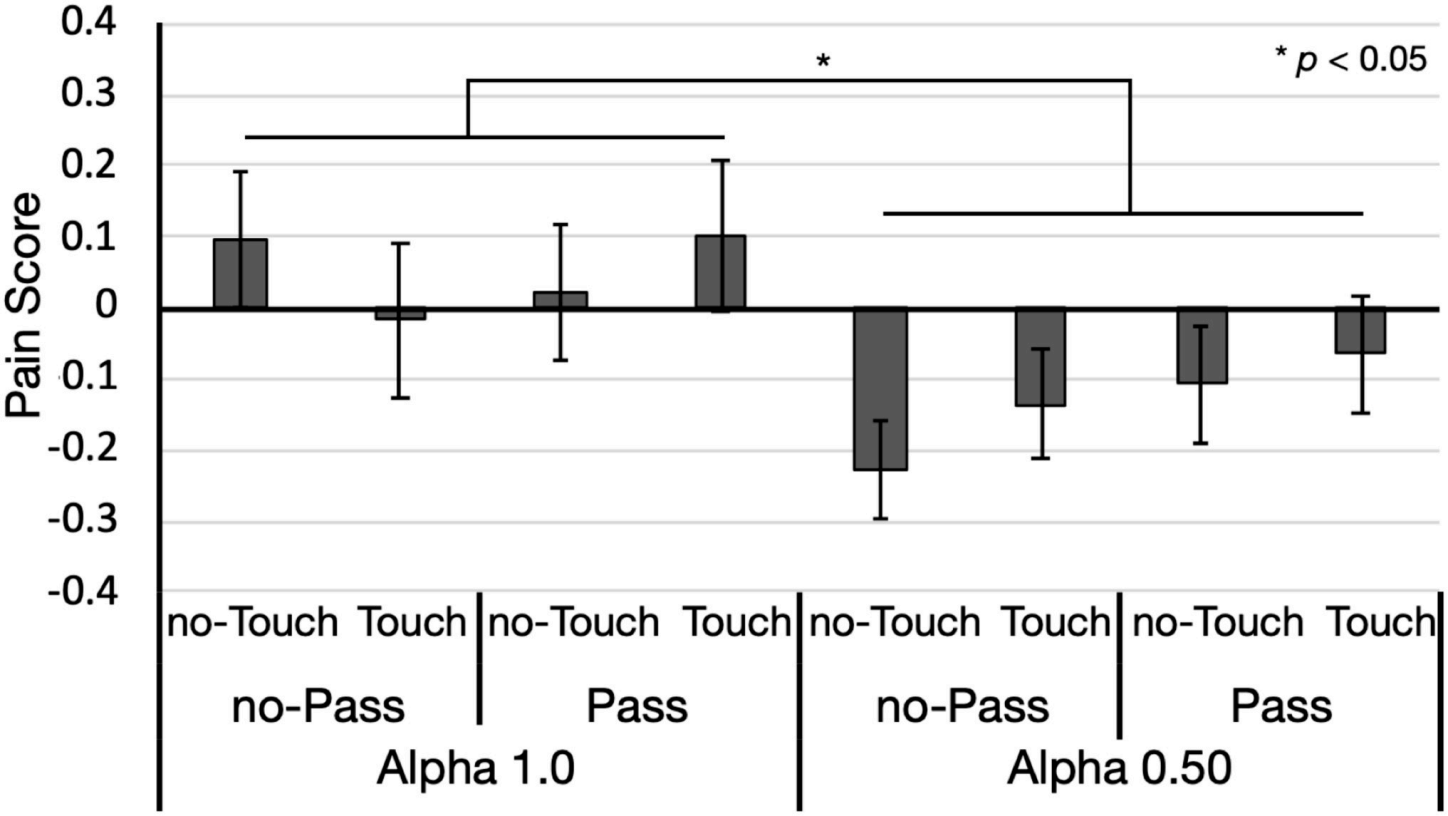
Pain scores for each condition in Experiment 2 (*n* = 26). Each bar represents the average pain scores. When the evaluation was identical to baseline, then the pain score reached 0. Moreover, the baseline was measured without the HMD prior to all experimental conditions. A negative score indicates that perceived pain decreased more than the baseline evaluation, whereas a positive score denotes that perceived pain increased more than the baseline evaluation. On the x-axis, data are grouped according to the alpha-value (1.0 or 0.5), pass-through (with and without), and touch (with and without) manipulations. Error bars denote standard errors.

Fig 5 displays the pain scores for each condition. If the score was zero, then the intensity of perceived pain under a given condition was identical that evaluated with full ownership sense and no transparency without the HMD. A three-way ANOVA revealed the significant main effect of the alpha-value manipulation (*F*(1, 25) = 5.691, *p* = 0.025, $\eta_{G}^{2}=0.039$). The pain score decreased at alpha value = 0.25. All other effects were non-significant (*F*s (1, 25) < 2.750, *p*s > 0.110, $\eta_{G}^{2}s<0.005$).

Considering its empirical uses, examining whether perceived pain increased, decreased, or remained from the baseline, which is measured without the HMD, is important. Toward this end, we compared the pain score for each condition to a score of 0. The reason for this notion is that if the pain intensity did not differ from baseline, then the pain score should be 0. The pain score was significantly less than 0 at alpha value = 0.5 with the touch manipulation and without the pass-through manipulation (*t*(25) = 3.255, *p* = 0.003, *d* = 0.638). In other conditions, the pain score did not significantly differ from 0 (*t*s (25) < 1.755, *p*s > 0.090, ds<0.344).

The results indicated that the alpha-value manipulation influenced the pain score, which also mainly affected body ownership, transparency, and intangibility. The pass-through manipulation exhibited a strong effect on the scores for ownership and intangibility but not the pain score. Moreover, the alpha-value manipulation influenced anxiety to a certain extent. However, the pass-through manipulation exhibited a stronger effect on anxiety. The results suggested that transparency exerted the strongest effect on the intensity of perceived pain.

We calculated the correlations between the scores for each feeling and the pain score (Table 4). The strongest negative correlation was observed between transparency and pain score (*r*(181) = −0.268, *p* < 0.001). The other feelings exerted significant but weaker correlations to the pain score compared with transparency, that is, intangibility and anxiety were negatively correlated to pain (intangibility: *r*(181) = −0.175, *p* = 0.018; anxiety: *r*(181) = −0.167, *p* = 0.024), whereas ownership and stiffness exhibited a positive correlation with pain score (ownership: *r*(181) = 0.154, *p* = 0.038; stiffness: *r*(181) = 0.168, *p* = 0.023). The correlation among the feelings, except for stiffness, was strong.

**Table 4:** Correlation coefficients between the feelings and pain score in Experiment 2 (*n* = 26).

	Ownership	Transparency	Intangibility	Anxiety	Stiffness
Transparency	−0.545***				
Intangibility	−0.652***	0.704***			
Anxiety	−0.641***	0.370***	0.583***		
Stiffness	−0.039	0.053	−0.021	−0.058	
Pain	0.154*	−0.268***	−0.175*	−0.167*	0.168*

* *p* < 0.05.

** *p* < 0.01.

*** *p* < 0.005.

Additionally, the pain score was smaller than that at baseline when alpha value = 0.5 with the touch manipulation and without the pass-through manipulation. As shown in Fig 4, under this condition, the scores for ownership and transparency were relatively high. This result suggested that transparency decreased perceived pain, particularly when the participants felt that the observed limb belonged to their body. In contrast, the effect of transparency seemed to be weakened with the decrease in body ownership.

## Discussion

This study used AR technology to modify body representation. By manipulating the value of the alpha channel, the participants observed their forearm and hand becoming transparent. Additionally, we used a virtual stick to pass through the limb. The manipulations successfully altered the body representation but decreased the body ownership of the participants at the same time.

The modifications of body representation influenced subjective pain intensity. The manipulation of the alpha value induced a decrease in perceived pain and an increase in the feeling of transparency. Our results suggest that the feeling of their limb becoming transparent favored the decrease in perceived pain. Additionally, this effect was stronger with a high level of body ownership.

### Change in body representation

We succeeded in changing the participants’ body representation, that is, they believed that their body became a transparent one. However, the alpha-value manipulations exhibited an effect not only on body representation (i.e., transparency and intangibility) but also body ownership and, under several conditions, anxiety. Additionally, the pass-through manipulation increased the feeling of intangibility but decreased body ownership. These manipulations enabled the participants to experience phenomena that do not occur in real life. In other words, the wooden stick never passed through the arm, whereas the arm does not become transparent in the real world. Therefore, the unnatural phenomenon happening to the limb may have decreased the sense of body ownership, although we hypothesized that high levels of ownership will be retained using the AR technique.

Interestingly, the pass-through manipulation did not influence body ownership at alpha value = 0.25 where we provided the most transparent appearance. This finding suggests that the participants considered the phenomenon of an object passing through a very transparent limb to be natural. A similar tendency for anxiety further supported this idea. Intangibility increased with the alpha-value manipulation even without the pass-through manipulation. In other words, the features evoked by visual appearance, namely the intangibility evoked by the decrease in alpha value, may be applied to the observed limb. As a result, the stick passing through the limb felt natural. Evidently, the following interpretation was possible: the participants perceived the passing of the stick as unbelievable and could not feel it actually passing through. However, we considered this scenario unlikely because intangibility increased with the pass-through manipulation in the same condition. In Experiment 2, alpha value = 0.50 may not have generated sufficient transparency to induce intangibility. Therefore, the score for anxiety may have displayed a complex tendency, such that the possibility exists that the conflict between the touch and pass-through manipulations increased anxiety.

### Body representation and pain

Previous studies revealed three possible sources of the analgesic effect: observing one’s body, immersion (attention) in a virtual environment, and body state. The subsequent text discusses the possible involvement of the sources of the observed analgesic effect.

#### Observing one’s body

Previous studies demonstrated that observing one’s body decreased perceived pain [31–33]. The participants also felt less pain when they perceived that an artificial or virtual body/body part belonged to their body (i.e., high ownership; [19–21, 34–39]). This effect implies a negative correlation between pain and body ownership. However, in the current study, the alpha-value manipulation decreased the scores for ownership and perceived pain. Thus, the analgesic effect triggered by observing one’s body does not explain the results of the experiment.

However, this analgesic effect may have occurred in all conditions because the participants observed their limb throughout the experiments. The pain score in Experiment 2 did not increase from baseline values, which were obtained without the HMD, that is, when the participants observed their bodies with full ownership. The analgesic effect was undetected due to the lack of a control condition, that is, the participants did not observe their limbs. The investigation of the analgesic effect of the observation of one’s body is beyond the scope of the study but may constitute an important topic of investigation in the future.

#### Immersion in a virtual environment

Evidence exists that immersive and interactive virtual reality environments can decrease perceived pain [40, 41]. For example, Hoffman et al. [40] demonstrated this effect in the treatment of burn injuries. The study found that the analgesic effect was stronger when the VR environment was more immersive. In other words, the patients allocated more attention to the immersive environment and were distracted from the painful situation in the real world.

Our experiments did not measure the feeling of immersion. However, the score for body ownership may reflect this feeling. The alpha-value manipulation rendered the situation less plausible, which resulted in low levels of body ownership. This result suggested that the world became less immersive. However, in Experiment 2, the pass-through manipulation influenced body ownership but not the pain score. When the limb was touched without pass-through manipulation, a certain level of ownership and immersion were retained but did not influence the pain score. Therefore, the attentional factor discussed by previous studies [40, 41] did not offer a suitable explanation for the current results. Additionally, the participants observed their limb in the experiments. If they focused their attention to the scene being observed, that meant that they focused on their biological limbs. Therefore, they were not distracted from the painful stimulus. Nevertheless, we assume that the attentional factor may be related to the analgesic effect in the current study, which will be discussed later.

#### Body state

Previous studies also demonstrated the effect of body state on pain perception. For instance, the sensation of pain was increased for an injured virtual limb [42, 43], whereas the pain threshold to heat was decreased for a virtual limb with red skin [10]. In addition, many researchers investigated the effect of size [12, 13]. At the level of the nervous system, the GSR to an approaching threatening stimulus changed when the participants believed that their limb was made of marble based on sound manipulation [4]. In the current experiment, three scores represented the mental body state, namely, transparency, intangibility, and stiffness. We added stiffness following Senna et al. [4]. However, the manipulations in the experiment did not target stiffness, which remained the same under all conditions. The results indicated that transparency was the feeling that is most suitable for explaining perceived pain. As the participants’ feeling of transparency decreased, perceived pain decreased. However, intangibility did not increase the analgesic effect.

The sense of body ownership was deemed to modulate the effect of transparency. Indeed, the decrease in perceived pain was greater with high levels of body ownership. We hypothesized that transparency decreased perceived pain, whereas low levels of body ownership decreased the analgesic effect induced by observing the limb, which resulted in high levels of perceived pain. In addition, the possibility exists that body representation was less modified in participants with low levels of body ownership because they less considered the observed limb as their own, which decreased the effect of transparency on perceived pain. In a previous study, the level of pain was low for an injured virtual limb if no body illusion procedure was conducted [43]. Romano et al. [13] illustrated that only when a virtual body was embodied can body size influence skin conductance response. Thus, further investigation is required to reveal the relationship between body ownership and change in the mental body model.

### Transparency and pain

Prior to the experiments, we predicted that making the participants’ limbs transparent would decrease perceived pain, because it made them feel that nothing can touch them (intangible). However, the results of the experiments demonstrated that the intangible feeling is unable to explain the change in perceived pain. Then, why did making the limb transparent decrease the intensity of perceived pain? Matamala-Gomez et al. [22] found that making the body transparent among CRPS type-I patients decreased their chronic pain rating. The authors argued that this analgesic effect may be related to disturbances in body representation [22]. However, all participants in the current experiments were healthy adults and may have had normal body representation.

From the results of the experiments, we are unable to conclude why transparency decreased perceived pain. However, one possible effect of transparency may be the decrease in the feeling of “existing in the (real) world.” In other words, the participants felt as if they were disappearing as a result of the alpha-value manipulation. This feeling denotes presence but differs from the abovementioned immersion in the virtual world. In the context of the study, presence pertains to presence in the real world. Newport and Gilpin [44] led their participants to feel as if their limb disappeared and no longer existed in the world. They demonstrated that such a feeling decreased the SCR to threat. Comparing with our method, the authors used a more sophisticated strategy for multisensory integration. However, the scenarios were similar to ours in which the limb seemed to disappear. Apparently, the participants understood that if we continued to decrease the alpha value, their limb would no longer be observable. Therefore, the participants may have perceived a decrease of their presence in the real world.

Moreover, the symptoms of depersonalization include a feeling of being “cut off from the world” and “I have to touch myself to make sure that I have a body or a real existence,” which are feelings related to the perception that one’s body has disappeared. Researchers proposed that patients with depersonalization had a high pain threshold [45, 46]. According to studies about the hand-disappearing trick and depersonalization, the decrease in alpha value induced the feeling of disappearing from the world, which distracted participants from the real environment. As a result, perceived pain may have decreased from when they observed their non-transparent limb. This phenomenon is also deeply related to body ownership, which will be discussed in the subsequent text.

Alternatively, Martini et al. [11] argued that making the virtual limb transparent does not point to a clear relationship with pain threshold. This difference may have been caused by a different type of stimulus. Specifically, we consider the time duration of the stimulus as one of the important components for determining the analgesic effect. The transparency of the limb may exert the largest effect at the first moment of stimulus. Body representation was updated from moment to moment according to the given stimulus. Therefore, if the stimulus is provided for a certain duration similar to that of Martini et al. [11], then haptic feedback may alter body representation or expectation for pain and, in turn, the analgesic effect within the same period.

### Ownership and pain

Despite the fact that the decrease in body ownership impaired the analgesic effect in many previous studies, body ownership and perceived pain in the present study decreased with the alpha-value manipulation. We used the AR technique with the objective of retaining high levels of body ownership despite the alpha-value manipulation. Although the ownership score decreased when we changed the alpha value, the use of the AR technique may be related to the positive relationship between body ownership and intensity of perceived pain. Without the alpha-value manipulation, the participants exhibited very high levels of body ownership. The participants observed all processes of their limb becoming transparent. For this reason, they were completely aware of their limb despite the feeling that it did not belong to them and led to the illusion of the limb disappearing.

In terms of such a decrease in body ownership, their biological limb (body) felt as if it did not belong to them, which previous studies termed “disownership” [47]. The abovementioned studies by Newport and Gilpin [44] on depersonalization [45, 46] referred to the same phenomenon or symptom (i.e., disownership). In situations where disownership occurred along with the feeling of being cut off from the world, the perceived pain of participants or patients decreased [44–46]. Similarly, in this study, the intensity of perceived pain may have decreased along with disownership and the feeling of their limb disappearing. Thus, studies should consider the different roles of body ownership on artificial objects and biological bodies in terms of pain perception. In this regard, we should further consider that when the participants perceived body ownership over artificial objects, then body ownership over their biological body decreased [48].

### Limitation

This study has its limitation. The first is that the baseline rating in the VAS was low for several participants, although we confirmed that they felt pain. Thus, we should further confirm that subjective pain from the baseline stimulus will not influence the analgesic effect in future works. Given that the experimenters in previous studies that used a heat threshold ceased the increase in stimulus temperature when the participants felt pain, those in the current study were given a stronger stimulus. Therefore, the current experiments demonstrated that the pain stimulus was felt as a weak painful stimulus. However, according to our procedure, whether the stimulus that invoked weak pain was perceived as a non-pain stimulus similar to previous studies remains unclear, which also requires examination.

The second limitation is the experimental procedure. We instructed the participants to evaluate pain intensity after removing the HMD. The reason for this step is to decrease their physical fatigue. Additionally, our method to realize the transparent limb (i.e., color extraction) was not suitable for VAS evaluation. Therefore, physiological indices that capture online responses to the pain stimulus can solve this problem.

Additionally, we did not control for the length of the duration of observing the limb. Under conditions with alpha value = 1.0, without pass-through manipulation, without movement manipulation, and without touch manipulation, we only omitted the manipulation and did not add any alternative manipulation or duration of observation. We deem that this omission did not influence our conclusion, that is, even conditions whose time duration is nearly the same displayed a different tendency in the pain score. Thus, a more controlled experiment will support this notion.

## Conclusion

This study intended to alter body representation by manipulating the appearance of the limb and investigated its influence on pain perception. We made the participant perceive their limbs as transparent using AR technology. The results indicated that the participants perceived less intense pain with the increased transparency of the limb, which suggests the influence of body representation on pain perception. The decrease in perceived pain was greater when a high level of body ownership was retained. Further investigations are required to elucidate the mechanisms involved in the role of body representation and body ownership on pain perception.
